# Variations in CTAC batches from different suppliers highly affect the shape yield in seed-mediated synthesis of gold nanotriangles

**DOI:** 10.1038/s41598-023-50337-4

**Published:** 2024-08-23

**Authors:** Ekaterina Podlesnaia, Amarildo Hoxha, Sreevalsan Achikkulathu, Athulesh Kandathikudiyil Antony, Jerestine Philomina Antony, Kathrin Spörl, Andrea Csáki, Matthias Leiterer, Wolfgang Fritzsche

**Affiliations:** 1 Department of Nanobiophotonics, Leibniz Institute of Photonic Technology (Leibniz-IPHT), Member of the Leibniz Research Alliance – Leibniz Health Technologies, 07745 Jena, Germany; 2 Thüringer Landesamt für Landwirtschaft und Ländlichen Raum (TLLLR), 07743 Jena, Germany

**Keywords:** Nanoparticles, Synthesis and processing

## Abstract

The rapidly developing miniaturization in numerous fields require low-demanding but robust methods of nanomaterial production. Colloidal synthesis provides great flexibility in product material, size, and shape. Gold nanoparticle synthesis has been thoroughly studied, however, recent reports on mechanistic insights of crystal formation have been hindered by the numerous procedures and parameter optimization works. With every new study, scientists fill another blank space on the map of understanding anisotropic growth and find out the critical parameters. In the current work, we highlight the choice importance for surfactant supplier in achieving the gold nanotriangle formation. We systematically study the variation in the shape yield when utilizing five batches of cetyltrimethylammonium chloride (CTAC) from varied suppliers. Using analytical techniques, we search for deviations causing such variation, e.g. different impurity content. We found only a marginal effect of iodine contamination on the studied system, excluding this factor as decisive in contrast to what was proposed earlier in the literature, and leaving the high dependency of the yield to originate from yet unknown reagent characteristics. A deeper understanding of these factors would provide highly effective protocols lowering the reagent consumption and increasing the accessibility of nanomaterials manufactured in a sustainable manner.

## Introduction

The rapidly emerging field of optics and bioanalytics requires the development of nanomaterial production, which should be low demanding but provide a high yield and control over the product characteristics^1,2^. Namely, the application of localized surface plasmon resonance (LSPR) in sensorics is highly dependent on the fabrication of demanded metal nanoparticles^3–12^. Among a great number of techniques, colloidal synthesis has been known as an affordable but flexible method to manufacture nanoparticles of various materials, sizes, and shapes^13–18^. For decades the kinetics of such processes has been studied following two general approaches: one-pot (seedless) and multi-step (seed-mediated)^18–29^. For both, the nucleation of metal atoms forming the first clusters is followed by their growth. In seedless synthesis, these phenomena occur in the same medium with the possible overlap at a certain time stage^30^. This may cause a low reproducibility or inhomogeneity of the product. Separation of the nucleation and growth steps by controlling the kinetics provides the seed-mediated approach with higher accuracy and robustness^23^.

However, lately, the mechanistic insights of crystal formation were hindered by the numerous synthetical procedures and parameter optimization reports. As a consequence, a number of seemingly similar protocols leading to the formation of various shapes were reported^27,28,31,32^. Meanwhile, there is no self-consistent procedure, which allows controlling the particle morphology by varying the reaction conditions but starting from a single batch of spherical seeds. Such obstacles in a full understanding of anisotropic growth are caused by the numerous parameters that need to be taken into account and balanced carefully. Among these factors are reagent characteristics, ambient conditions (e.g. temperature), total volume, mixing and diffusion limitations, pH, and ionic strength. With every new study, scientists fill another blank space on the map of understanding nanoparticle synthesis. For instance, we recently reported the crystallinity of seeds being highly dependent on the mixing intensity and affecting the nanotriangle growth^33^.

The reagent characteristics represent a distinguished category of synthesis parameters. They may vary by the concentration, purity grade, long-time stability, and the role played in the process. In a typical gold nanoparticle synthesis, the conversion of Au^3+^ ions into metal Au^0^ is achieved by using a reducing agent in aqueous medium. A highly active reagent, such as NaBH_4_, is required to initiate the nucleation to produce the first Au^0^ atoms. These atoms form initial clusters (nuclei) coalescing and slowly growing into larger particles, which are utilized as seeds in further nanoparticle growth^34^. The gold surface acts as catalyst lowering the redox potential of HAuCl_4_ and allowing the use of milder reducing agents, such as ascorbic acid, for the subsequent growth of larger particles^35^. To achieve controlled nucleation and provide stable colloids, stabilizing agents are utilized^36,37^. Together with additives, such as halides or silver ions, they are also used in the last growth step to control the resulting morphology^38–44^. Such shape control is commonly related to the selective adsorption of surface active species on specific crystal planes of nucleating centers^45,46^. Ionic surfactants, such as cetrimonium halides, are widely used as shape-directing agents^47^. In the aqueous phase, the head group of the surfactant molecule adsorbs on the charged nanoparticle surface leaving the hydrocarbon tail insolubilized. It leads to the adsorption of another surfactant layer resulting in the formation of a close-packed bilayer^48,49^.

A number of previous works were done on studying the influence of the surfactant concentration, counter ion, and alkane chain length, on the yield of the target morphology^17,50,51^. But only a few studies point to the chemical manufacturer as one of the key factors for successful shape control. For instance, Millstone and co-workers have reported the contamination of CTAB batches with iodine leading to the formation of nanotriangles instead of nanorods^52^. Using inductively coupled plasma mass spectrometry (ICP-MS), they show that only certain CTAB batches contain I^–^. Those samples, as well as the purified CTAB with the introduced optimal concentration of iodide ions, favor the growth of nanoparticles into a triangular shape. X-ray photoelectron spectroscopy (XPS) revealed I^–^ was bound to the (111) crystal facets of Au nanotriangles, but not to the surface of rods and spheres. Based on these observations the authors suggest that normally a CTAB bilayer attaches to the entire Au surface by the electrostatic force, which does not provide a preferential growth resulting in isotropic shapes. If iodine ions are present in the growth medium, they preferentially bind to the (111) facet of a nanoparticle inducing the growth on the remaining open (110) and (100) facets^38^. The authors also highlight that the binding energies of halide ions adsorption on gold surfaces scale with polarizability, I^–^ > Br^–^ > Cl^–^, and crystal facet, (111) > (110) > (100)^41,53,54^, which affects the kinetics of growth and hence the final product morphology. Moreover, the present halides are introduced into the CTA-X-[AuX_2_]^−^ complexes and changing their solubility and reduction potentials, both of which decrease in the order [AuCl_2_]^−^  > [AuBr_2_]^−^  > [AuI_2_]^−^. Therefore the addition of iodide to CTAC will slow down the reduction rate, which is known to be beneficial for the formation of such shapes as triangular plates (nanotriangles) and octahedra^40,46,55^.

Meanwhile, Smith and co-authors showed the use of cetyltrimethylammonium bromide (CTAB) batches from varied suppliers drastically affects the yield of gold nanorods^51^. While the distinction in synthesis results is demonstrated, the determining factor remains unclear as no significant differences were found between the CTAB samples. The authors emphasize the importance of such study especially for newly established systems. Without reasoning, an unfortunate choice of the reagent supplier yielding no anisotropic growth may lead to the decision of abandoning the research in the field of interest. We experienced similar issues when started working on the synthesis of gold nanotriangles (AuNTs), which implements CTAC and sodium iodide as shape-directing agents^28^. Depending on the bottle of sufractant picked up from the lab shelf, the color of the resulting colloids could vary from clear blue (typical for the high-purity AuNT dispersions) to purple (indicating the presence of isotropic shapes formed as by-products). A batch from another supplier resulted in a wine red color with only a slight blue shade meaning a very low content of nanotriangles.

In the current work we systematically study the variation in the shape yield when utilizing different batches of CTAC from five suppliers in each synthesis step. Our primary research question centers on understanding the key factors influencing the product quality. Nanoparticle colloids were evaluated using ultraviolet–visible (UV–VIS) spectroscopy, transmission and scanning electron microscopy (TEM, SEM). The surfactant samples were tested with analytical techniques such as coupled plasma mass spectrometry (ICP–MS) and ion chromatography (IC) in order to trace the contamination level of iodine and bromide respectively. Additionally, we studied how sensitive the present synthesis is to the addition order and concentration of iodide ions. With this study, we aim to provide valuable insights into nanoparticle synthesis, leading to the development of highly effective and robust procedures.

## Results and discussion

**Scheme 1:**
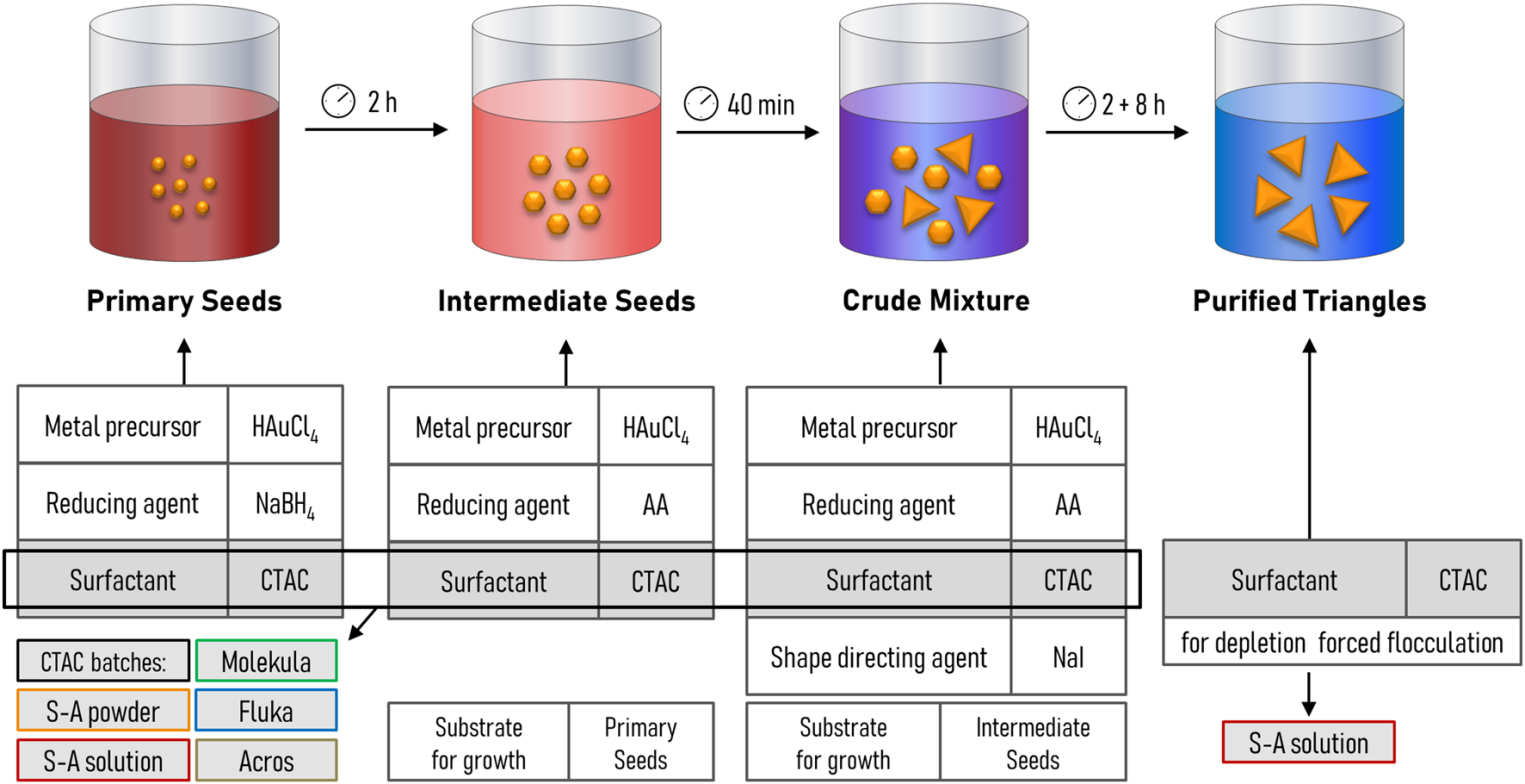
Three-step synthesis of gold nanotriangles. Primary and intermediate seeds are aliquoted into growth solutions of the corresponding synthesis step. The incubation at room temperature follows the intervals according to our earlier work on time-optimization^56^.

Our work is based on the earlier established three-step synthesis of gold nanotriangles^28,56^ as described in Scheme 1. Firstly, tiny gold clusters of a few nanometers called primary seeds (PS) are formed. Next, they are stabilized by the growth into larger intermediate seeds (IS). Finally, sodium iodide is used as shape-directing agent to obtain so-called crude mixtures, containing nanotriangles along with other by-product shapes. Implementation of depletion forced aggregation allows the collecting of a purified colloid of nanotriangles. We next discuss the results obtained by varying the CTAC batch at each step of the procedure. The CTAC solution from Sigma-Aldrich was selected for purification in all of the experiments due to no growth occurring at this step. Using it in liquid form directly available from the manufacturer eases the workflow by avoiding preparation of the concentrated CTAC stock solution.

### Primary and intermediate seeds formation in varied CTAC solutions

**Figure 1:**
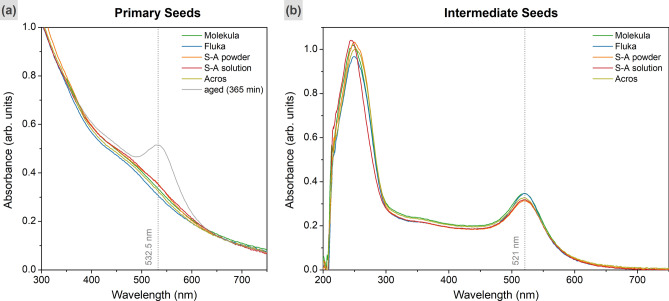
UV–VIS spectra of primary (**a**) and intermediate (**b**) seeds obtained in varied CTAC solutions. Grey line shows the LSPR peak corresponding to the aged PS. The vertical dashed lines indicate the peak positions at 532.5 nm (aged PS) and 521 nm (IS).

Five CTAC batches from varied suppliers were utilized to form the primary and intermediate seeds. The UV–VIS spectra of PS samples show a typical slope without an LSPR peak due to their miniscule sizes of ca. 2 nm (Fig. 1)^28,33,56^. The peak at 521 nm emerges in the spectra of IS as their sizes increase to 10 nm. Besides the intensity variation, there is no clear distinction in curve shape for all of the analysed samples.

**Figure 2:**
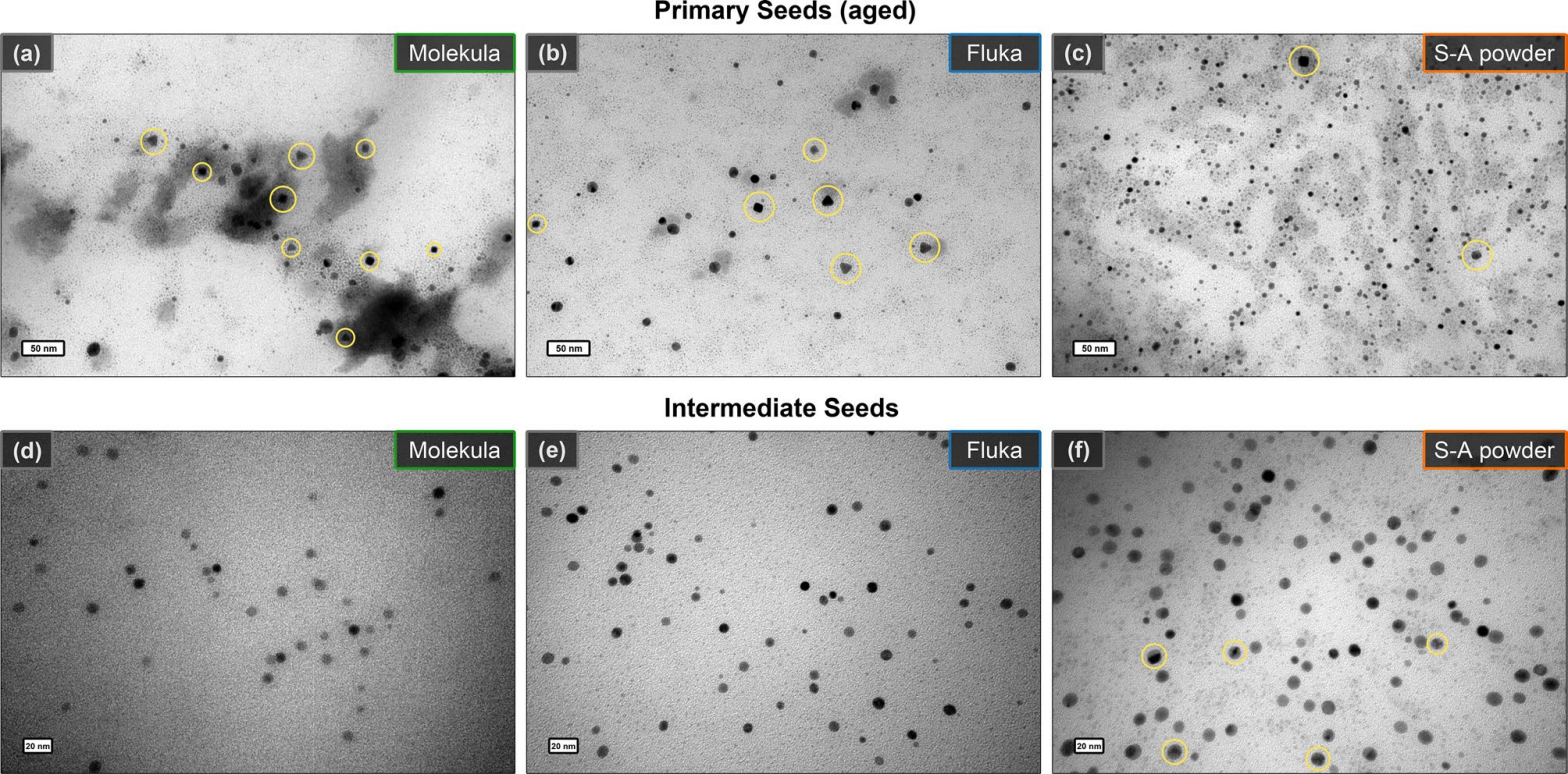
TEM images of primary (**a**–**c**) and intermediate (**d**–**f**) seeds (scale bars are 50 and 20 nm, respectively). The captions indicate utilized CTAC batches (from Molekula, Fluka, and Sigma-Aldrich powder). The anisotropic shapes (cubes, triangles etc.) in aged PS samples are highlighted in yellow. Variation in image contrast of IS indicates the presence of planar defects, which are especially visible in (**f**) as highlighted by yellow.

Interestingly, the TEM micrographs revealed the formation of anisotropically shaped nanoparticles in aged primary seeds (Fig. 2, Fig. S1). Along the spherical particles, some cubes and triangles were observed, although there is no shape-directing agent added at this step. Therefore, it would be fair to assume that the formation of anisotropically shaped agglomerates could be caused by some impurities in the used surfactant, which would also affect further growth steps. Nevertheless, the TEM of intermediate seeds shows only spheres of ca. 10 nm in diameter with planar defects in their structure, which are essential for nanotriangle formation^45,57^. The presence of isotropic shapes is likely due to the fast process of gold reduction during their growth. On opposite, the aging of primary seeds happens slowly enough for symmetry breaking events to appear^40,46,55^.

### Nanotriangle growth in varied CTAC solutions

**Figure 3:**
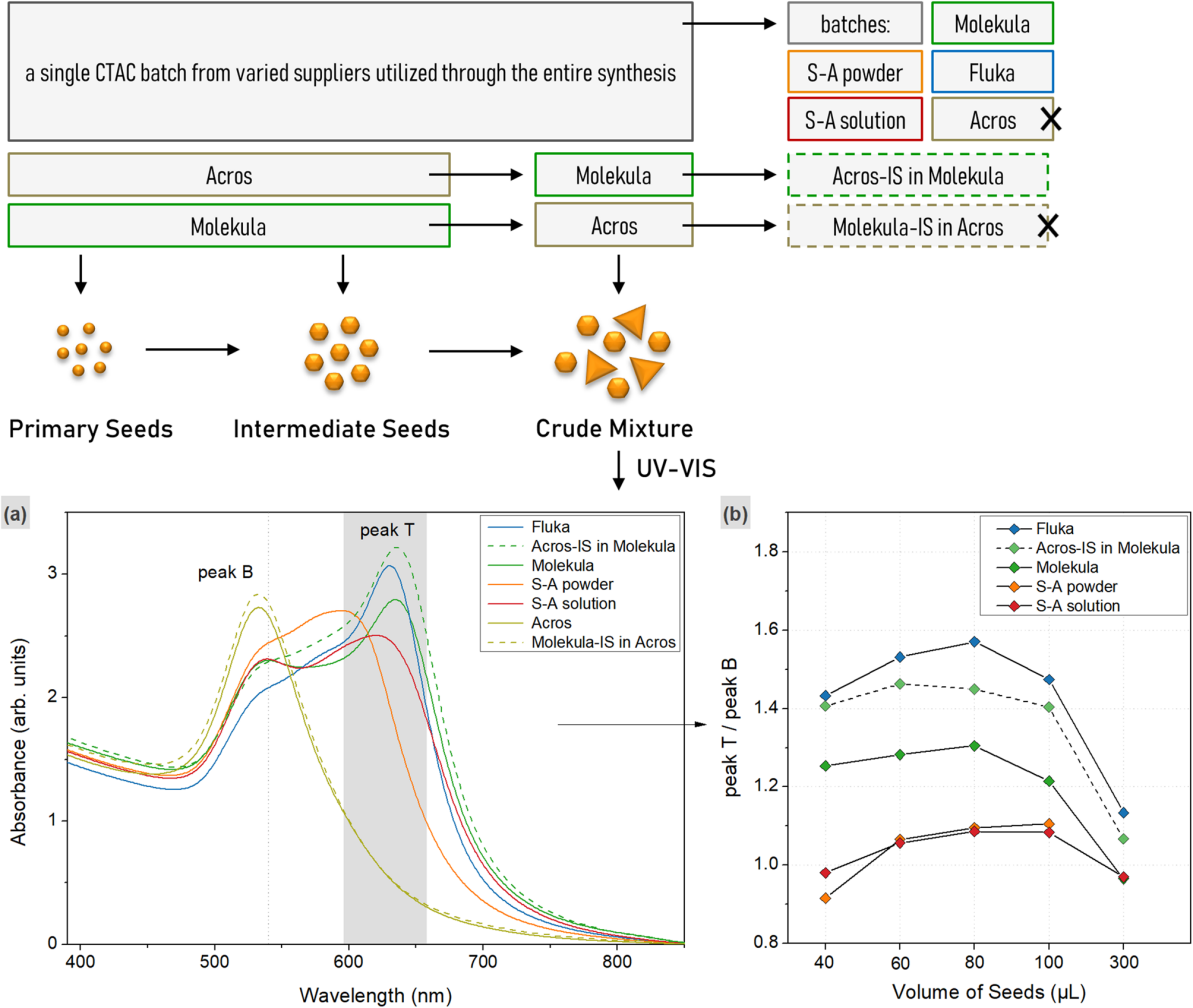
Reaction scheme on the top indicates the experiment design and the color code for the derived samples. UV–VIS spectra (**a**) of the crude mixtures obtained from 100 μl of IS in varied CTAC solutions as indicated in the legend (**b**). The dotted lines indicate the positions of the peaks related to triangles (peak T at 596–658 nm) and by-products (peak B at 540 nm). The ratios of the peak intensities (right) demonstrate the shape yield (the X-axis indicates the utilized volume of IS in μL).

Each of the prepared IS was used to form the crude mixtures using the corresponding CTAC batch but keeping all of the other reaction parameters the same. Typically, the reaction mixtures demonstrated a color evolution from pink to purple and eventually blue of slightly various shades. In contrast, the samples prepared with CTAC from Acros turned wine red with a light blue shade indicating a poor triangle yield. In the UV–VIS spectra, the LSPR peaks in a higher wavelength range of ca. 596–658 nm indicate the presence of triangles (peak T), while the peaks at ca. 540 nm and lower wavelengths are related to the by-products of other shapes (peak B). The curves revealed various ratios of peak intensities, and hence the shape yield, depending on the surfactant supplier (Fig. 3, Fig. S2, Table S1). The series of samples prepared in Fluka and Molekula CTAC show the highest content of triangles. The lowest shape yield was achieved by using the batch supplied by Sigma-Aldrich in both powder and solution forms. A single peak corresponding to only isotropic shape formation, or a very poor triangle yield, was observed for the series of samples derived in Acros CTAC.

We carried out an additional test on whether the use of an appropriate surfactant batch is critical at each of the reaction steps or has the most influence during the crude mixture formation. For this, two parallel series of synthesis were carried out. At first, intermediate seeds formed in CTAC from Molekula were introduced into the growth solution prepared using a batch from Acros. The resulting crude mixtures and purified nanotriangles were labelled as “Molekula-IS in Acros”. The UV–VIS spectra for all of the samples show a single LSPR peak at 533 nm (peak B), and are similar to the samples which were derived utilizing only Acros CTAC at each synthesis step (Fig. 3, Fig. S2). No triangle formation (absence of peak T) could be detected meaning that the choice for suitable CTAC plays the critical role at the latest step of the process. To confirm this, the reversed series “Acros-IS in Molekula” was produced, for which IS formed in CTAC from Acros were applied for anisotropic growth in the batch from Molekula. The high intensity of peak T indicates triangle formation, showing that independently on the CTAC batch used during the formation of primary and intermediate seed, the anisotropic growth is mainly affected by CTAC sample utilized at the latest growth step. Surprisingly, the ratio of the peak intensities for “Acros-IS in Molekula” series is even higher than for the samples obtained from the synthesis using only Molekula batch throughout the entire process (Table S1).

**Figure 4:**
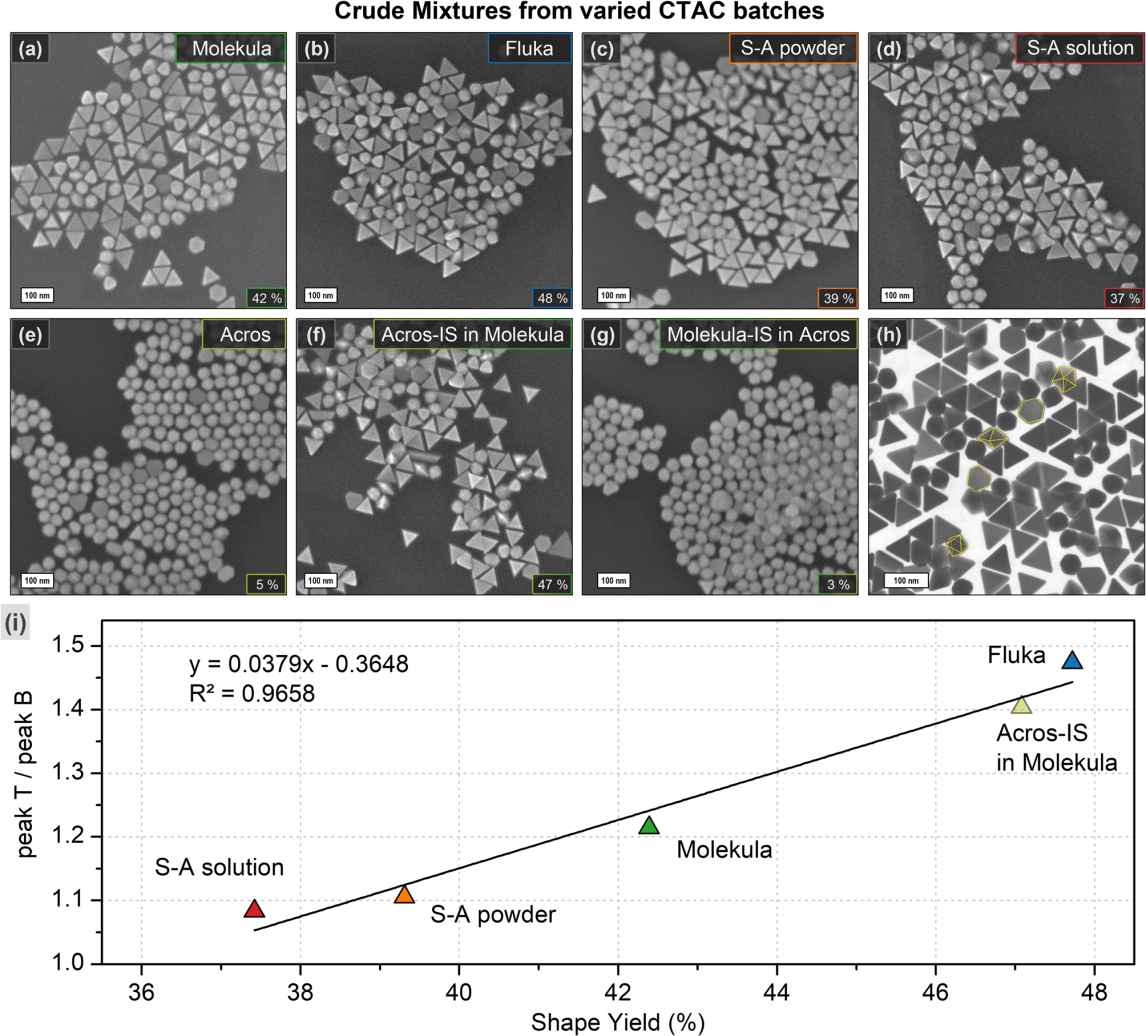
SEM images of the crude mixtures (**a**–**g**) formed using 100 μL of intermediate seeds in CTAC solutions prepared from varied batches as indicated by the captions. The scale bars are 100 nm. The numbers in the lower right corners indicate the triangle yield (%) based on 1150 counts on average for each sample. STEM micrograph of a representative sample (**h**) demonstrates the by-product structure as highlighted in yellow. The graph (**i**) shows the correlation between the ratios of triangle to by-product peak intensities (UV–VIS in Fig. 3) and the shape yield (SEM) in the crude mixtures formed from varied CTAC batches.

The SEM micrographs of crude mixtures reveal the various ratios of the triangle to by-product content proving the conclusions from the UV–VIS spectra analysis (Fig. 4). The highest impurity content was found in samples formed in CTAC from Acros, only 3–5% of particles have a well-defined triangular shape. The solution from Sigma-Aldrich provided a yield of 37–39%, which comes in agreement with the 45% previously reported by Szustakiewicz and co-authors who used the same product (25 wt% in water)^28^. However, the highest triangle content of 42–48% was derived using the batches from Molekula and Fluka. The statistical data correlates well with the findings based on the ratio of the peaks in the UV–VIS spectra proving that the latter can be used as an express method to estimate the variation in the shape yield (Fig. 4i). To ensure that obtained results are reproducible, we compared the spectra of crude mixtures on different synthesis dates. Although the intensity of the curves vary slightly, the ratio of nanotriangle to by-product peaks remain comparable. Hence, general dependency of the yield on the CTAC batch from specific supplier is consistent (Fig. S2). STEM imaging shows the by-products to be of such morphologies as octa-, deca- and other high degree polyhedra along with triangular bipyramides, hexagonal or truncated triangular plates. These shapes are known to be the product of seeds with multiple or single twin defects, similar to triangular plates (nanotriangles)^45,57^.

**Figure 5:**
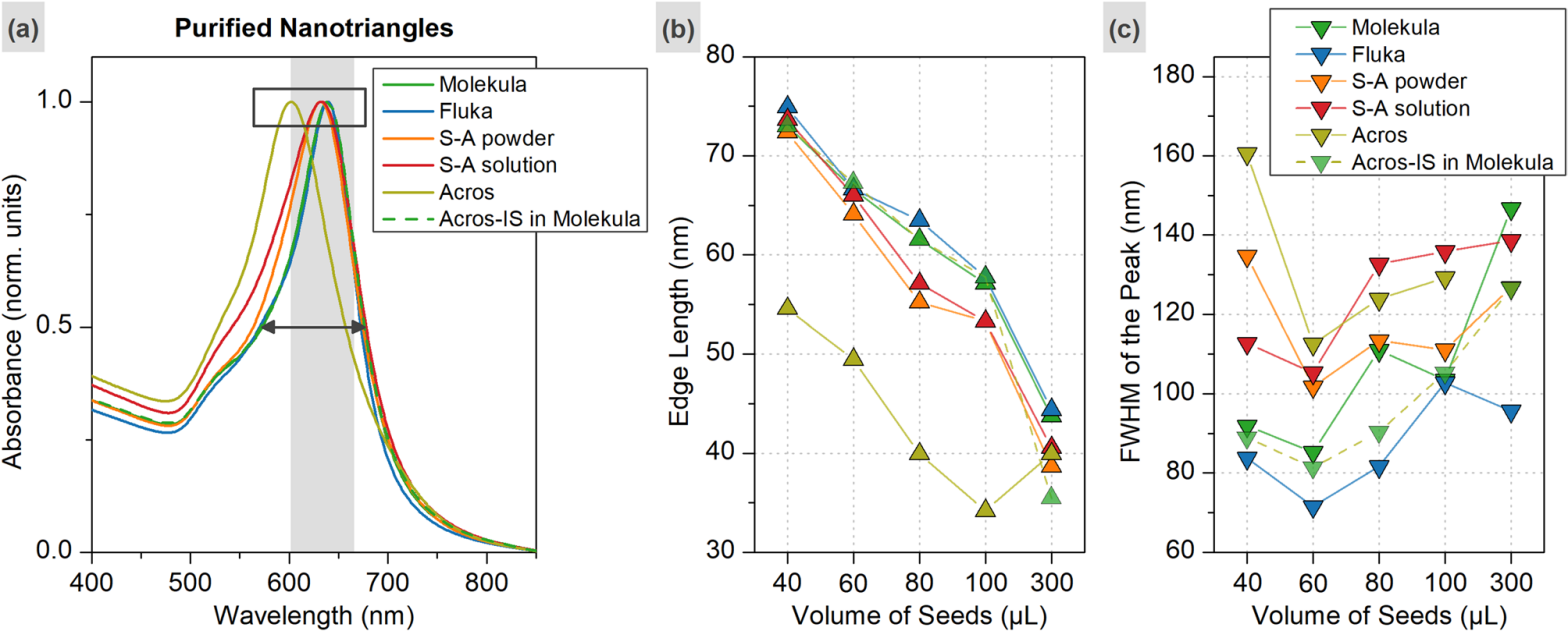
Normalized UV–VIS spectra of purified triangles obtained from 100 μl of IS in varied CTAC solutions as indicated in the legend (**a**). The average edge length of nanotriangles estimated based on the LSPR peak position (**b**), and the FWHM of the peak (**c**), were used to assess the characteristics of the purified products (the X-axes indicate the utilized volume of IS in μL).

Next, the crude mixtures were purified using depletion-forced aggregation. The resulting gold nanotriangle colloids were analyzed with UV–VIS spectroscopy (Fig. 5, Fig. S4). The average size (edge length of nanotriangle) was calculated based on the LSPR peak position as described in the previous reports^28,33,56^. The full width at the half maximum (FWHM) of the absorption peak was used to assess the size distribution. The largest and the most homogeneous triangles, which are characterized by lowest FWHM values, were derived using Molekula and Fluka batches. Notably, these series also demonstrated the highest shape yield as discussed above. A very low quantity of smaller and more polydisperse triangles was collected from the series synthesized with Acros batch. The numerical data can be found in Table S2.

### Analysis of CTAC batches and variation of iodide concentration in growth solutions

**Figure 6:**
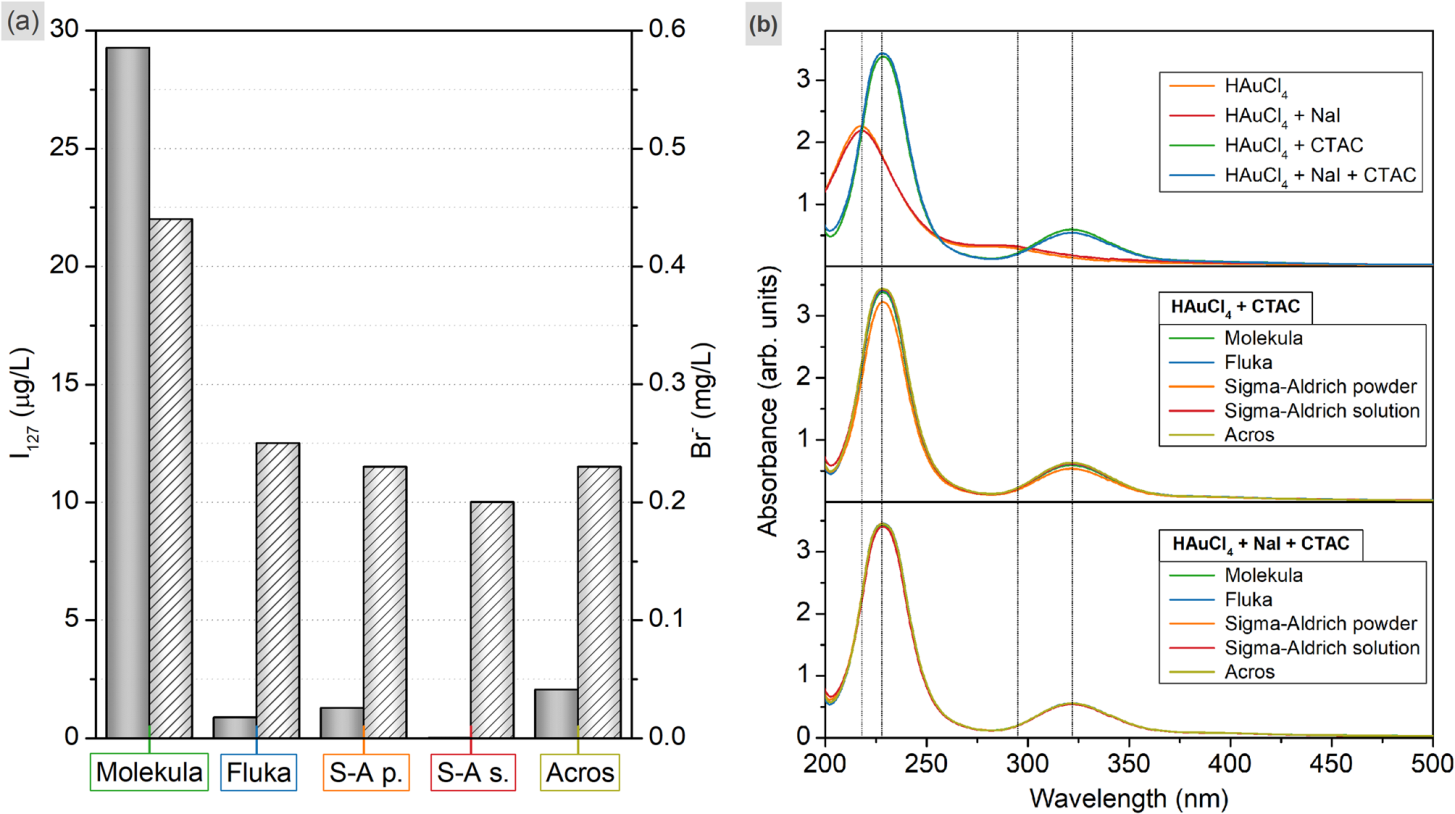
(**a**) Iodine (grey bars) and bromide (white patterned bars) concentrations detected in varied 0.02 M CTAC solutions using ICP-MS and IC respectively. (**b**) UV–VIS spectra of aqueous solutions containing 0.59 mM HAuCl_4_, 73 μM NaI, and 46.36 mM varied CTAC as indicated in the legend (the probes are fivefold diluted to avoid a saturation in intensity).

The formation of anisotropic shapes in the aged samples of primary seeds together with the higher yield of nanotriangles for Molekula and Fluka CTAC may be caused by non-controlled halide contamination of the batches. A similar assumption was made in the earlier reported studies of the gold nanorod synthesis being dependent on the CTAB supplier^51^. By controlling the iodide concentration in such a system the nanotriangle yield was tuned from a low amount up to the predominant part of the product^52^. Keeping this in mind, we decided to study utilized CTAC batched with highly sensitive analytical techniques. ICP-MS revealed the highest content of I_127_ in the sample from Molekula (29.26 μg/L), which could explain the higher shape yield (Fig. 6a, Table S3). However, the remaining batches, including Fluka, show significantly lower values (0.87–2.05 μg/L), the deviation of which is likely caused by the value proximity to the limit of quantification (0.1 µg/l), and the measurement uncertainty (15% for liquid phase) under the conditions studied. Recalculation of these quantities to the 50 mM CTAC as used for anisotropic growth returns 0.20–0.58 μM of iodide, which is significantly lower than 73 μM typically introduced into the growth solution. The bromide concentration was determined with IC, and was found to be the highest again in Molekula CTAC. Other samples demonstrate lower but similar values (0.20–0.25 mg/L is similar to the background value of 0.2 mg/L, which is not subtracted from the reported results). As no explicit trend was found in halide concentration, we performed semi-quantitative ICP-MS measurements using the "Total Quant" method. It is a high-quality analysis to determine the approximate concentrations of 78 elements providing their fingerprint in a sample (Table S4, Fig. S5). Although, this method could potentially provide reasoning for the synthesis results, the elemental distribution of the CTAC solutions is complex and varies from batch to batch which does not allow drawing a straightforward conclusion on which deviations play a key role. To the best of our knowledge, cetrimonium halides are produced from petroleum or plant oils^58^. The differences in content of studied CTAC batches might be caused by the variety of raw materials and method used for their synthesis. However, such information is commonly not reported by the suppliers, which hinders a rational understanding of impurity origin and their role in nanoparticle formation.

In the growth solution the gold precursor typically interacts with the surfactant molecules, which influences its optical properties. The UV–VIS spectrum of pure HAuCl_4_ shows the single band at 218 nm and a shoulder at ca. 295 nm (Fig. 6b). With the addition of CTAC the color of chloroauric acid turns from transparent to turbid yellow with the change in the bands appearing at 228 and 322 nm due to the formation of CTA-Cl-[AuCl_4_]^−^ complex ions. The curves obtained for all of the samples demonstrate the same features not showing the formed complexes being affected by the batch variation. Moreover, there are no visible differences in the spectral characteristics of HAuCl_4_ in the presence or absence of the iodide ions in the growth solutions. Apparently, the used concentration of 73 μM is yet low to affect the stability of [AuCl_4_]^−^ ions and not enough to facilitate an exchange reaction. However, it is still possible that the minor non-detectable changes in studied conditions are still crucial in the process of crystal developments^38^.

**Figure 7:**
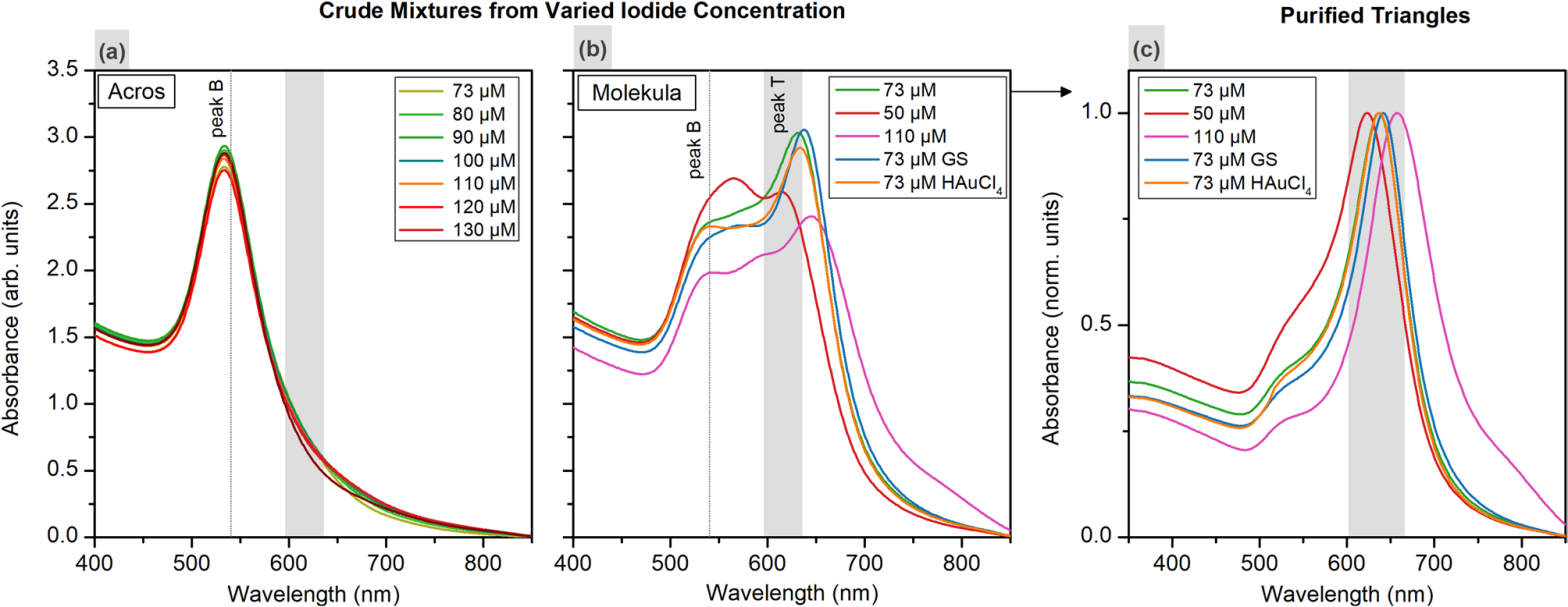
UV–VIS spectra of the crude mixtures formed in Acros ((**a**) 80 μL of IS) and Molekula ((**b**) 100 μL of IS) CTAC solutions with the varied I^–^ concentration and its addition order, and normalized spectra for the corresponding purified samples (**c**). The dotted lines indicate previously determined peak positions for the samples from the same volume of IS.

In their work, Millstone and co-authors, could reproducibly drive the reaction forming the target morphology by deliberately adjusting iodide concentration in pure CTAB^52^ The growth solutions containing 50 μM of I^–^ predominantly yielded triangles (ca. 65% before purification). At higher concentrations (> 75 μM), more rounded disk-like particles were produced. We tested whether, in the case of our system, the iodide concentration could be tuned to an optimal value in order to induce anisotropic growth in CTAC from Acros, which yielded no nanotriangles earlier when following a standard procedure working with final concentration of 73 μM iodide (Fig. 3). For this, intermediate seeds were introduced into growth solutions with iodide concentration varied from 80 to 150 μM. Formed crude mixtures demonstrate a single peak at ca. 530 nm in UV–VIS spectra meaning that only isotropic by-product shapes, and no nanotriangle, were obtained (Fig. 7a). A similar experiment was conducted but using Molekula CTAC throughout the synthesis as it provided high nanotriangle yield. The iodide concentration was varied from 50 and 110 μM, and yet the crude mixtures show two LSPR peaks, which indicates the formation of nanotriangles along with by-products (Fig. 7b). The shape yields are genreally lower than at the seeming optimal iodide concentration of 73 μM. Also, the LSPR peak positions, and hence the dimensions, of purified nanotriangles were affected by the variation of iodide concentration (Fig. 7c). Nevertheless, these experiments show that the current synthesis is not as sensitive to the halide contamination level as was considered in earlier works on similar procedures.

**Figure 8:**
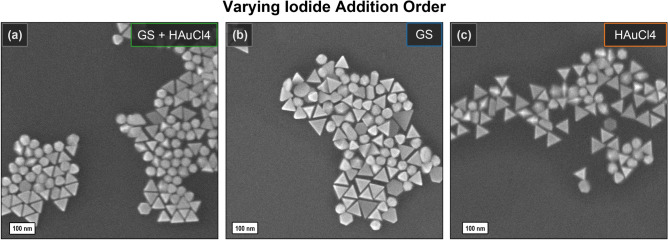
SEM of crude mixtures obtained with 100 μL of IS in Molekula CTAC varying the iodide addition order: standard procedure ((**a**) GS + HAuCl_4_), to growth solution ((**b**) GS) and to HAuCl_4_ premix (**c**) only.

Additionally, we studied the role of the iodide addition order. In a typical synthesis, 40 μL of NaI is added to both a growth solution containing CTAC and intermediate seeds, and a premix with HAuCl_4_ acting as a gold precursor. According to the literature, this is required for the pre-incubation of iodide ions on IS surface causing the symmetry breaking^28^. However, it is not justified why another half of the amount is premixed with tetrachloroauric acid, which is afterwards injected to initiate the growth. When mixing both, the pale yellow color of concentrated gold precursor turns orange which is apparently due to the partial exchange of chlorine to iodine in [AuCl_4_]^–^ complex, leading to a decrease in its solubility. This would slow down the reduction kinetics, favoring the formation of nanotriangles as a morphology covered with lower-energy surface facets^40,46,55^. To find out the critical step for iodide addition, we produced two parallel batches of crude mixtures by adding the full amount of iodide into growth solution or HAuCl_4_ only. The latter sample revealed a lower intensity of the major peak in the UV–VIS spectrum (orange line in Fig. 7b,c). The crude mixture formed from the growth solution with the full amount of iodide (blue line) contained slightly larger triangles but lower impurity content compared to the standard procedure. The SEM images revealed well-defined triangular shape in all of the samples (Fig. 8).

## Conclusions

The current work emphasizes the need for thorough parameter study in the nanoparticle synthesis. Besides the known impurities, the reagents carry some yet unknown characteristics, variation of which drastically affect the resulting product. We assume that these deviations might be caused by the differences in raw materials and production methods of surfactants, which are commonly not disclosed by the manufacturers. Although such factors cannot be controlled by individual researchers, it is important to keep them in mind as critical, especially when establishing novel methods of nanoparticle synthesis. We demonstrated the seed-mediated synthesis of gold nanotriangles being highly dependent on the surfactant manufacturer, which was earlier observed by other groups for various anisotropic morphologies. However, our findings contrast with the previous works, showing the current method being not as sensitive to halide impurities as it was claimed for similar procedures. The batch from Acros resulted in a very poor anisotropic growth. However, the use of IS obtained in it but further grown in Molekula CTAC lead to the significant increase of the shape yield, which is also ca. 10 percentage points higher than from the commonly used reagent from Sigma-Aldrich. Moreover, changing the iodide addition slightly increased the content of triangles compared to when following the original procedure. A deeper mechanistic understanding of the shape anisotropy formation would allow lowering the reagent consumption by increasing the product yield and quality. This brings a perspective of increasing the accessibility of nanomaterials manufactured in a sustainable manner.

## Experimental section

### Chemicals and materials

All utilized chemicals were obtained commercially and used without further purification. Tetrachloroauric(III) acid trihydrate (HAuCl_4_ ∙ 3H_2_O ≥ 99.5%), L( +)-ascorbic acid (AA,  ≥ 99%) were purchased from Carl Roth GmbH & Co KG (Karlsruhe, Germany). Sodium borohydride (NaBH_4_, 99.99%), sodium iodide (NaI,  ≥ 99.5%) were purchased from Sigma-Aldrich Chemie GmbH (Steinheim, Germany). Hexadecyltrimethylammonium chloride (CTAC) was obtained from four different suppliers: Molekula Group GmbH, powder, > 99% (Munich, Germany); Fluka Chemie GmbH, powder 98% (Buchs, Switzerland); Sigma-Aldrich Chemie GmbH, powder ≥ 98.0%, and solution 25% w/w in water (Steinheim, Germany); Acros Organics, powder 99% (Fisher Scientific GmbH, Schwerte, Germany). The solutions were prepared using Milli-Q water (EQ 7000; Merck KGaA, Darmstadt, Germany). Prior to use, all glassware and magnetic stirrers were washed with aqua regia (caution: aqua regia is highly toxic and corrosive) and rinsed thoroughly with Milli-Q water.

### Synthesis and purification of gold nanotriangles

Three-step synthesis of gold nanotriangles was conducted following the earlier reported time-optimized procedure^56^. The CTAC from varied suppliers was utilized in parallel experiments to study an impact on the shape yield. Obtained mixtures were purified using depletion forced aggregation. A complete precipitation was achieved using the following CTAC concentrations: 450, 200, 175, 125, and 100 mM applied to the crudes mixtures derived from 300, 100, 80, 60, and 40 µL of intermediate seeds respectively.

### Characterization techniques and instrumentation

The gold nanoparticle colloids were characterized with ultraviolet–visible (UV–VIS) spectroscopy utilizing Thermo Fisher NanoDrop OneC (Waltham, MA, USA) and JASCO V-670 UV–VIS–NIR (Easton, PA, USA) spectrophotometers.

Scanning transmission electron microscopy images (STEM) were acquired using a FEI Helios NanoLab G3 UC (Hillsboro, OR, USA). Transmission electron microscopy (TEM) was conducted using a JEOL JEM 1400 (Akishima, Japan). Scanning electron microscopy (SEM) images were taken using a JEOL FE-SEM JSM-7900F (Akishima, Japan). The probes for imaging were prepared following the modified literature technique^56,59^. An amount of 750 μL of as-prepared crude mixtures was centrifuged and re-dispersed in 1 mL of 0.1 mM CTAC followed by another centrifugation cycle. The final sample was re-dispersed in 120 μL of 0.1 mM CTAC ensuring the optimal surfactant concentration. Then 2.0–2.5 μL were deposited on a Formvar coated copper grid (Plano) or on a silicon substrate for SEM, and air-dried.

Analisys of CTAC batches was carried out using the 0.1 M solutions diluted in 1:5 ratio. Inductively coupled plasma mass spectrometry (ICP-MS) was conducted using ICP-MS NexION 350XX (Perkin Elmer LAS GmbH, Rodgau, Germany), with addition of 0.2 ml NH_4_OH to the each sample, standard and background measurements. Ion chromatography (IC) was performed with IC type 881 compact IC pro (Metrohm, Herisau, Switzerland). Additionally, ICP-MS was used to perform the semi-quantitative “Total Quant” measurements using 0.1 M CTAC solutions diluted in 1:10 ratio and a comparative sample (water standard). The instrument was calibrated with Mg, Rh and Pb, so mass distributed over the periodic table.

The spectral data manipulation was performed using OriginLab software (OriginLab Corporation, Northampton, MA, USA). The electron microscopy images were analyzed with ImageJ software (National Institutes of Health and University of Wisconsin, USA).

## Supplementary Information

Supplementary Information.

## Data Availability

The datasets used and/or analysed during the current study available from the corresponding author on reasonable request.
