# Supplementary material for: Variations in CTAC batches from different suppliers highly affect the shape yield in seed-mediated synthesis of gold nanotriangles
Source: Sci Rep. 2024 Aug 23;14:19610. doi: 10.1038/s41598-023-50337-4 (PMC11344135; doi:10.1038/s41598-023-50337-4)
Supplement: Supplementary file 1 — Supplementary Information. [file 41598_2023_50337_MOESM1_ESM.docx]

**Supporting Information**

**Variations in CTAC batches from different suppliers highly affect
the shape yield in seed-mediated synthesis of gold nanotriangles**

**Ekaterina Podlesnaia^1,^ *, Amarildo Hoxha^1^, Sreevalsan Achikkulathu^1^, Athulesh Kandathikudiyil Antony^1^, Jerestine Philomina Antony^1^, Kathrin Spörl^2^, Andrea Csáki^1^, Matthias Leiterer^2^, and Wolfgang Fritzsche^1, *^**

^1^ Leibniz Institute of Photonic Technology (Leibniz-IPHT), Member of the Leibniz Research Alliance – Leibniz Health Technologies, Department of Nanobiophotonics, Jena, 07745, Germany

^2^ Thüringer Landesamt für Landwirtschaft und Ländlichen Raum (TLLLR), Jena, 07743, Germany

**Figure S1.** TEM images of primary (top) and intermediate (bottom) seeds obtained in CTAC solutions from Sigma-Aldrich solution (left) and Acros (right).

**

**

**Figure S2.** UV–VIS spectra of the crude mixtures from varied CTAC solutions (as indicated in the graph titles). The numbers in legends indicate the utilized volume of IS in μL. The dotted lines indicate the positions of peaks related to by-products (peak B at 540 nm) and nanotriangles (peak T at 596–658 nm).

**Table S1.** The positions of the peaks (λ_max_) related to triangles and the ratios of peak intensities
(peak T / peak B) in the UV–VIS spectra of the crude batches (derived from Figure S2).

| **V (IS), μL** | **CTAC batch** | **λ_max_ (peak T), nm** | **peak T / peak B** |
| --- | --- | --- | --- |
| 300 | Molekula | 609 | 0.965573 |
|  | Fluka | 601 | 1.133594 |
|  | Sigma-Adlrich powder | — | — |
|  | Sigma-Adlrich solution | 603 | 0.970036 |
|  | Acros-IS in Molekula | 605 | 1.067073 |
| 100 | Molekula | 635 | 1.214656 |
|  | Fluka | 631 | 1.474036 |
|  | Sigma-Adlrich powder | 596 | 1.105675 |
|  | Sigma-Adlrich solution | 620 | 1.083328 |
|  | Acros-IS in Molekula | 636 | 1.403753 |
| 80 | Molekula | 642 | 1.304656 |
|  | Fluka | 638 | 1.570893 |
|  | Sigma-Adlrich powder | 599 | 1.095722 |
|  | Sigma-Adlrich solution | 627 | 1.085741 |
|  | Acros-IS in Molekula | 640 | 1.449677 |
| 60 | Molekula | 647 | 1.28249 |
|  | Fluka | 645 | 1.531609 |
|  | Sigma-Adlrich powder | 613 | 1.065700 |
|  | Sigma-Adlrich solution | 629 | 1.056598 |
|  | Acros-IS in Molekula | 647 | 1.463018 |
| 40 | Molekula | 658 | 1.253136 |
|  | Fluka | 656 | 1.432859 |
|  | Sigma-Adlrich powder | 625 | 0.915784 |
|  | Sigma-Adlrich solution | 642 | 0.980763 |
|  | Acros-IS in Molekula | 655 | 1.406217 |

**Figure S3.** Exemplar SEM images for the shape yield calculations in the crude mixtures formed from varied CTAC batches.





**Figure S4.** Normalized UV–VIS spectra of the purified triangles from varied CTAC solutions (as indicated in the graph titles). The numbers in legends indicate the utilized volume of IS in μL. The dotted lines indicate the peak positions (602–666 nm).

**Table S2.** The peak positions (λ_max_) and the full width at the half maximum (FWHM, calculated using the integration tool in OriginLab) in the UV–VIS spectra of the purified triangles. The edge length (L) was calculated based on the linear function: L = 0.6361 × λ_max_ − 348.76 ^28,33,56^.

| **V (IS), μL** | **CTAC batch** | **λ_max_, nm** | **Edge Length, nm** | **FHWM, nm** |
| --- | --- | --- | --- | --- |
| 300 | Molekula | 617 | 43.7 | 147 |
|  | Fluka | 618 | 44.4 | 96 |
|  | Sigma-Adlrich powder | 609 | 38.6 | 127 |
|  | Sigma-Adlrich solution | 612 | 40.6 | 139 |
|  | Acros | 611 | 39.9 | — |
|  | Acros-IS in Molekula | 604 | 35.5 | 127 |
| 100 | Molekula | 638 | 57.1 | 103 |
|  | Fluka | 639 | 57.7 | 103 |
|  | Sigma-Adlrich powder | 632 | 53.3 | 111 |
|  | Sigma-Adlrich solution | 632 | 53.3 | 136 |
|  | Acros | 602 | 34.2 | 129 |
|  | Acros-IS in Molekula | 639 | 57.7 | 105 |
| 80 | Molekula | 645 | 61.5 | 111 |
|  | Fluka | 648 | 63.5 | 82 |
|  | Sigma-Adlrich powder | 635 | 55.2 | 113 |
|  | Sigma-Adlrich solution | 638 | 57.1 | 133 |
|  | Acros | 611 | 39.9 | 124 |
|  | Acros-IS in Molekula | 645 | 61.5 | 90 |
| 60 | Molekula | 653 | 66.6 | 85 |
|  | Fluka | 653 | 66.6 | 71 |
|  | Sigma-Adlrich powder | 649 | 64.1 | 102 |
|  | Sigma-Adlrich solution | 652 | 66.0 | 105 |
|  | Acros | 626 | 49.5 | 113 |
|  | Acros-IS in Molekula | 654 | 67.3 | 81 |
| 40 | Molekula | 663 | 73.0 | 92 |
|  | Fluka | 666 | 74.9 | 84 |
|  | Sigma-Adlrich powder | 662 | 72.4 | 135 |
|  | Sigma-Adlrich solution | 664 | 73.6 | 113 |
|  | Acros | 634 | 54.5 | 161 |
|  | Acros-IS in Molekula | 663 | 73.0 | 89 |

**Table S3.** Iodine and bromide concentrations detected in varied 0.02 M CTAC solutions using ICP-MS and IC respectively.

| **CTAC batch** | **ICP–MS** | | **IC** | |
| --- | --- | --- | --- | --- |
|  | **I 127, μg/L** | **blank** | **Br^–^, mg/L** | **blank** |
| Molekula | 29.84 | 0.59 | 0.44 | 0.2 |
| Flluka | 1.53 | 0.67 | 0.25 | 0.2 |
| Sigma-Adlrich powder | 1.85 | 0.58 | 0.23 | 0.2 |
| Sigma-Adlrich solution | 0.50 | 0.52 | < 0.20 | 0.2 |
| Acros | 2.63 | 0.58 | 0.23 | 0.2 |

**Table S4.** Semi-quantitative "Total Quant" overview obtained using ICP-MS. The values show approximate concentrations of 78 elements (ppb) in the 0.01 M CTAC solutions from varied suppliers.

| **Analyte** | **Concentration (ppb)** | | | | |
| --- | --- | --- | --- | --- | --- |
|  | Molekula | Fluka | S-A powder | S-A solution | Acros |
| H | — | — | — | — | — |
| He | — | — | — | — | — |
| Li | 0.013 | 0.006 | 0.047 | 0.065 | 0.004 |
| Be | 0.002 | 0.002 | 0.003 | 0.016 | 0.005 |
| B | 0.859 | 2.446 | 0.872 | 1.09 | 0.895 |
| C | 525699.758 | 558488.54 | 507608.333 | 531296.401 | 543919.765 |
| N | 157185.583 | 169621.909 | 167066.25 | 173251.356 | 166902.104 |
| O | — | — | — | — | — |
| F | — | — | — | — | — |
| Ne | — | — | — | — | — |
| Na | 6.332 | 11.981 | 356.148 | 359.841 | 28.869 |
| Mg | 0.72 | 0.646 | 1.667 | 2.665 | 0.526 |
| Al | 0.505 | 0.393 | 1.101 | 3.047 | 0.342 |
| Si | 595.341 | 625.763 | 712.738 | 803.086 | 630.177 |
| P | 1.352 | 1.041 | 0.731 | 1.86 | 0.75 |
| S | 29174.203 | 30962.552 | 23289.338 | 25383.909 | 29606.371 |
| Cl | 166115.329 | 171726.807 | 166593.165 | 159132.828 | 172032.145 |
| Ar | 0 | 0 | 0 | 0 | 0 |
| K | 0 | 8.09 | 3.609 | 14.017 | 15.679 |
| Ca | 65.547 | 113.052 | 136.921 | 198.752 | 52.391 |
| Sc | 0.455 | 0.507 | 0.501 | 0.541 | 0.481 |
| Ti | 0.945 | 1.008 | 0.935 | 1.085 | 0.987 |
| V | 8.533 | 8.917 | 8.591 | 8.621 | 8.776 |
| Cr | 24.967 | 25.953 | 24.36 | 24.868 | 25.76 |
| Mn | 0.038 | 0.024 | 0.275 | 1.601 | 0.036 |
| Fe | 16.404 | 17.082 | 21.785 | 28.436 | 19.589 |
| Co | 0.003 | 0.003 | 0.009 | 0.02 | 0.006 |
| Ni | 0.206 | 0.238 | 0.346 | 0.557 | 0.201 |
| Cu | 0.035 | 0.08 | 0.338 | 0.338 | 0.041 |
| Zn | 1.531 | 0.556 | 0.167 | 0.443 | 0.345 |
| Ga | 0.005 | 0.004 | 0.059 | 0.275 | 0.012 |
| Ge | 0 | 0.002 | 0.001 | 0.001 | 0.001 |
| As | 1.872 | 1.908 | 1.794 | 1.818 | 1.94 |
| Se | 0.152 | 0.003 | 0.05 | 0.047 | 0.032 |
| Br | 20.106 | 4.743 | 4.043 | 3.498 | 4.931 |
| Kr | 0 | 0 | 0 | 0 | 0 |
| Rb | 0.001 | 0.004 | 0.003 | 0.011 | 0.005 |
| Sr | 0.007 | 0.018 | 0.027 | 0.031 | 0.008 |
| Y | 0 | 0 | 0 | 0 | 0 |
| Zr | 0 | 0.007 | 0.064 | 0.083 | 0.002 |
| Nb | 0 | 0 | 0.002 | 0.003 | 0.001 |
| Mo | 0 | 0.011 | 0.004 | 0.013 | 0.006 |
| Ru | 0 | 0 | 0 | 0 | 0 |
| Rh | 0 | 0 | 0 | 0.001 | 0 |
| Pd | 0 | 0.005 | 0.003 | 0.002 | 0.001 |
| Ag | 0.007 | 0.022 | 0.035 | 0.081 | 0.017 |
| Cd | 0.001 | 0.001 | 0.001 | 0.014 | 0.004 |
| In | 0.082 | 0.094 | 0.819 | 1.531 | 0.161 |
| Sn | 0.007 | 0.006 | 0.207 | 0.389 | 0.007 |
| Sb | 20 | 30.61 | 377.149 | 359.67 | 15.305 |
| Te | 0 | 0 | 0 | 0 | 0 |
| I | 93.001 | 37.986 | 47.037 | 176.996 | 26.717 |
| Xe | 0 | 0 | 0 | 0 | 0 |
| Cs | 0.006 | 0.005 | 0.048 | 0.112 | 0.019 |
| Ba | 0.163 | 1.858 | 0.645 | 1.126 | 0.978 |
| La | 0.001 | 0.002 | 0.014 | 0.016 | 0.001 |
| Ce | 0.001 | 0.002 | 0.029 | 0.168 | 0.002 |
| Pr | 0 | 0 | 0.001 | 0.002 | 0 |
| Nd | 0 | 0 | 0 | 0 | 0 |
| Sm | 0 | 0 | 0.001 | 0 | 0 |
| Eu | 0 | 0 | 0 | 0 | 0 |
| Gd | 0 | 0.001 | 0.057 | 0.349 | 0 |
| Tb | 0 | 0 | 0 | 0 | 0 |
| Dy | 0 | 0 | 0 | 0 | 0 |
| Ho | 0 | 0 | 0 | 0 | 0 |
| Er | 0 | 0 | 0 | 0 | 0 |
| Tm | 0 | 0 | 0 | 0 | 0 |
| Yb | 0 | 0 | 0.001 | 0.003 | 0 |
| Lu | 0 | 0 | 0 | 0 | 0 |
| Hf | 0 | 0 | 0.003 | 0.004 | 0 |
| Ta | 0 | 0.195 | 0.102 | 0 | 0.324 |
| W | 0.009 | 0 | 0.002 | 0.06 | 0.029 |
| Re | 0 | 0 | 0 | 0 | 0 |
| Os | 0 | 0 | 0 | 0 | 0 |
| Ir | 0 | 0 | 0 | 0 | 0 |
| Pt | 0.042 | 0 | 0 | 0 | 0 |
| Au | 0.517 | 0.485 | 0.785 | 2.358 | 0.8 |
| Hg | 0 | 0 | 0 | 0 | 0 |
| Tl | 0 | 0.001 | 0.003 | 0.014 | 0.005 |
| Pb | 0.009 | 0.013 | 0.034 | 0.055 | 0.014 |
| Bi | 0.071 | 0.059 | 1.499 | 3.056 | 0.156 |
| Th | 0.002 | 0.001 | 0.018 | 0.034 | 0.004 |
| U | 0.054 | 0.058 | 0.252 | 0 | 0.155 |





**Figure S5.** The concentration distribution for the selected elements based on the values in Table S4 obtained with the “Total Quant” method.


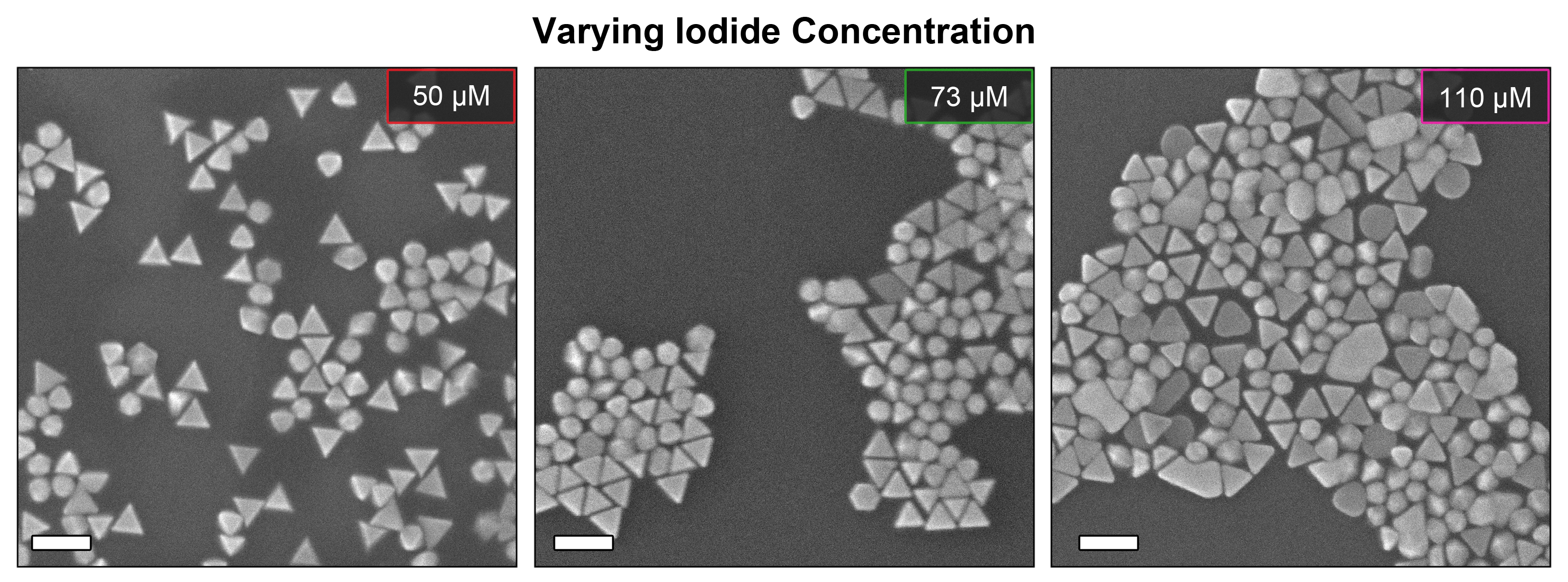


**Figure S6.** SEM of crude mixtures obtained with 100 μL of IS in Molekula CTAC varying iodide concentration: 50 (left), 73 (middle) and 110 (right) μM. The micrographs support UV–VIS data in Figure 8.
